# Assessment of stroke knowledge and awareness among primary healthcare providers: A cross-sectional survey from the Kezhou quality improvement in acute stroke care project

**DOI:** 10.3389/fpubh.2023.1136170

**Published:** 2023-03-08

**Authors:** Gui-Bing Ding, Qiang Sang, Hai-Ji Han, Xi-Ming Wang, Yan-Feng Wu

**Affiliations:** ^1^Department of Neurology, The Second Affiliated Hospital of Nanjing Medical University, Nanjing, China; ^2^Department of Neurology, The Affiliated Kezhou People's Hospital of Nanjing Medical University, Kezhou, China

**Keywords:** primary healthcare providers, stroke, awareness, emergency medical services, quality improvement, training strategies

## Abstract

**Objective:**

Acute stroke care is a highly complex type of emergency medical service (EMS) involving patient-centered care in a highly unpredictable and stressful environment with the help of several busy providers. The ability of primary healthcare providers (PHPs) to identify stroke onset early and further manage referrals to higher-level hospitals becomes critical.

**Methods:**

We conducted a cross-sectional survey about stroke knowledge and awareness among PHPs in China from September 2021 to December 2021. A total of 289 PHPs were divided into two groups, the stroke treatment window (STW) Aware group vs. the STW Unaware group according to their knowledge on the time window for acute ischemic stroke (AIS) management. Logistic regression analysis was performed to explore the predictors associated with knowledge of the time window for acute stroke management.

**Results:**

Of 289 PHPs surveyed during the study period, 115 (39.7%) participants were aware of the time window for stroke management and were in the STW Aware group, while 174 (60.2%) were in the STW Unaware group. Forty percent of PHPs in the STW Aware group were familiar with the secondary stroke prevention goal of <140/90 mmHg, compared with 27.01% in the Unaware group (*P* < 0.05). PHPs were not sufficiently aware of loss of consciousness also a symptom of stroke in two groups (75.7 vs. 62.6%, *P* < 0.05). A higher proportion of PHPs in the STW Aware group believed that thrombolysis was an effective treatment for AIS (96.5 vs. 79.9%, *P* < 0.01). Endovascular therapy is indicated for AIS was perceived by a higher proportion of PHPs in the STW Aware group than that in the Unaware group (62.6 vs. 6.9%, *P* < 0.01). Eighty percent of PHPs in the STW Aware group reported attending training on stroke management compared with 58.1% in the Unaware group (*P* < 0.01). Logistic regression results showed that the predictors of stroke knowledge and awareness among PHPs included sex (OR: 2.3, 95% CI, 1.2–4.6), received training (OR: 2.9, 95% CI, 1.60–5.1), and times of training per year (OR: 0.70, 95% CI, 0.6–0.9).

**Conclusions:**

PHPs present with a mild to moderate level of stroke management knowledge in northwest China. Strategies to help increase stroke knowledge and awareness among PHPs should be considered in order to help improve the stroke related health service system.

## Introduction

Stroke is the leading cause of death and disability in the general population, and the burden of stroke is increasing globally (1). According to the Global Burden of Disease (GBD) 2019, it is the major contributor of disability-adjusted life years (DALYs) (2). Furthermore, as the global population ages, the incidence, prevalence, and DALYs of stroke are expected to increase. Stroke has also become the leading cause of DALYs in China, with 45.9 million DALYs in 2019 (3). Over the past three decades, the crude death rate from stroke in China has been increasing rapidly, rising faster than any other country. In the coming years, China faces increasing challenges in reducing the morbidity, mortality and disease burden of stroke (3).

Early reperfusion therapy, including intravenous thrombolysis (IVT) and endovascular therapy (EVT), is the most effective evidence-based treatment for acute ischemic stroke (AIS). Despite strong guideline recommendations, however, only 10% to 20% of patients with AIS in China reach the hospital within 3 h (3, 4). Between 2019 and 2020, only 5.64% of patients were treated with IVT and 1.45% with EVT. These rates are much lower than in developed countries (4). The time from onset to reperfusion therapy affects mortality and favorable outcomes and should be considered a major goal in the management of patients with AIS (5). Emergency stroke care is highly complex and involves patient-centered care in a highly non-predictable and stressful setting with several busy providers, such as nurses, emergency physicians, radiologists, and neurointerventionists, coordinating with each other and requiring a high degree of functional operability. The American Heart Association (AHA) created what it called the 8Ds of Stroke Care, known as the Stroke Survival Chain, which are the primary steps family members, emergency and hospital staffs take in diagnosing and treating a stroke (6). Detecting stroke symptoms is the first link in the stroke survival chain. However, there is a lack of public awareness of stroke symptoms and the need for emergency medical care (7). In some countries, stroke patients prefer to seek general practitioners rather than emergency medical services (EMS), especially in many low- and middle-income countries (8).

Primary care providers (PHPs), who work in primary care facilities at the community level, are the foundation of China's tertiary care system after the 2009 health care reform (9). PHPs consist of physicians, nurses, lab technicians, imaging technicians, etc. There are two physician pathways representing becoming a general practitioner in China (10). The 3+2 pathway for assistant practitioners requires 3 years of college and 2 years of clinical training. Assistant practitioners perform protocol-limited clinical assignments under the direction and supervision of a national registered practitioner. This physician pathway is primarily in underdeveloped areas of China. The 5+3 pathway includes 5 years of bachelor's degree training in clinical medicine and 3 years of standardized residency training. PHPs provide diagnosis, initial treatment, and transfer of acute or chronic medical care to secondary or tertiary hospitals (11). When a stroke occurs, stroke patients generally consult their PHPs in rural areas of northwestern China (12). Therefore, the ability of PHPs to identify stroke onset early and further manage referrals to superior hospitals *via* EMS to stroke centers became essential (11). The present cross-sectional survey aims to further assess stroke knowledge and awareness among PHPs from the Kezhou Quality Improvement in Acute Stroke Care Project (KQI-ASC Project), in Kezhou, Northwest China, which may be used to provide appropriate training for PHPs in stroke management and as a reference for improving the stroke related health service system.

## Materials and methods

### Design and participants

Kezhou is located in Northwest China, with a total population of 0.622 million in 2020. It has 4 counties and 36 townships^1^. There is one tertiary general hospital, four secondary general hospitals, 37 primary healthcare service centers, and about 1,500 registered PHPs^2^. PHPs include physician, nurse, and others, such as laboratory technologist and imaging technologist. This was a cross-sectional study conducted among the PHPs in Kezhou from September 2021 to December 2021. All participants aged 18 and above were invited to participate in this study. The demographic characteristics of PHPs were showed in Table 1. The study was approved by the Institutional Review Board of the Affiliated Kezhou People's Hospital of Nanjing Medical University with the following IRB No: 2021-06-19. All participants provided their written consent prior to participation in the interview.

**Table 1:** Demographic characteristics of primary healthcare providers.

**Items**	**All (*n* = 289)**	**STW Aware group (*n* = 115)**	**STW Unaware group (*n* = 174)**	**χ^2^**	***P*** **-value**
**Sex**, ***n*** **(%)**				4.9	0.03
Male	66 (22.8)	34 (29.6)	32 (18.4)		
Female	223 (77.2)	81 (70.4)	142 (81.6)		
**Age**, ***n*** **(%)**				1.6	0.7
<20 years old	3 (1.0)	1 (0.9)	2 (1.2)		
20–40 years old	228 (78.9)	93 (80.9)	135 (77.6)		
41–60 years old	56 (19.4)	21 (18.3)	35 (20.1)		
>60 years old	2 (0.7)	0 (0)	2 (1.2)		
**Education**, ***n*** **(%)**				0.9	0.7
Secondary school	42 (14.5)	17 (14.8)	25 (14.4)		
Junior college	150 (51.9)	63 (54.8)	87 (50.0)		
Undergraduate	97 (33.6)	35 (30.4)	62 (35.6)		
**Current profession**, ***n*** **(%)**				1.4	0.5
Physician	136 (47.1)	58 (50.4)	78 (44.8)		
Nurse	136 (47.1)	52 (45.2)	84 (48.3)		
Others	17 (5.8)	5 (4.4)	12 (6.9)		
**Professional title**, ***n*** **(%)**				0.8	0.7
Senior	206 (71.3)	83 (72.2)	123 (70.7)		
Mid-level	59 (20.4)	21 (18.3)	38 (21.8)		
Junior	24 (8.3)	11 (9.6)	13 (7.5)		

STW, stroke treatment window.

### Specific questionnaire design

The questionnaire comprised three sections, including the demographic characteristics, the knowledge about risk factors, symptoms and acute management of stroke, and training on acute stroke management. The first section of this questionnaire collected the demographic data, including sex, age, education, current profession, and professional title. The second section was about the knowledge of risk factors, symptoms and acute management of stroke, including 9 questions. Risk factors for stroke included hypertension, high cholesterol levels, diabetes mellitus, obesity, smoking, and alcohol consumption. There were seven common symptoms of stroke onset, including weakness on one side of the body, facial weakness, speech impairment, impairment of balance, headache, visual impairment, and loss of consciousness. We also inquired about the F.A.S.T. Stroke Assessment (13). Among the items related to acute stroke management knowledge, the recommended targets for the lowering of blood pressure, glucose, best period for stroke treatment, whether thrombolytic or endovascular therapy indicated for acute ischemic stroke and pre-hospital managements in stroke patients were in accordance with published guidelines (14, 15). The third section investigated whether PHPs had received training, the frequency of training, and education strategies for stroke management.

All items in the questionnaire were discussed through both focus groups and panel reviews. The survey was administered anonymously by certified investigators following a formal protocol. We also pretested the survey with 50 PHPs in two hospitals and revised the questionnaire according to the feedback. The Cronbach's alpha of the total scale in this research was 0.82 and examined for testing the internal consistency reliability.

The questionnaires were distributed to all participants online *via* the Wenjuanxing platform (https://www.wjx.cn/) in four WeChat groups (the most widely used instant messaging software in China). These WeChat groups consist of some of primary care providers in Kezhou. The four WeChat groups had a total of about 720 primary care providers, with about 48% participating in the platform. Three hundred primary care providers responded to the survey, a response rate of 41.7%. We totally collected 300 questionnaires, 289 of which were used for research. Reasons for non-response included: incomplete demographic information (*n* = 6); and out of contact with participants (*n* = 5).

### Questionnaire data grouping

The purpose of this study was to make a statistical inference by analyzing the current difference in PHPs knowledge between aware and unaware groups according to the participants' perceptions about the stroke treatment window (STW). According to the time window for acute stroke treatment recommended by the Chinese Stroke Association guidelines for clinical management of cerebrovascular disorders, the optimal treatment time for acute ischemic stroke treatment is less than 4.5 h (15). Of 289 PHPs surveyed during the study period, 115 (39.7%) participants were aware of the time window for stroke management and were in the STW Aware group, while 174 (60.2%) were in the STW Unaware group.

### Statistical analysis

SPSS version 27 for Windows (IBM, Armonk, New York, USA) was used for data analyses. The data were presented as percentages (relative frequencies) for categorical data. Frequencies of categorical variables were compared using Pearson χ^2^-test when appropriate. To investigate factors associated with knowledge of the time window for acute stroke management, we used a binary logistic regression model. The degree of association between independent variables with the dependent variable was estimated by odds ratio (OR) with 95% confidence interval (CI). Statistical tests were two-tailed with a significance level of 5%.

## Results

### Demographic characteristics of primary healthcare providers

Of the 289 participants in the study, 39.7% were aware of the time window for stroke management. More than half of the PHPs were female (*N* = 223, 77.2%), while 22.8% were male. There was significant difference with respect to sex (*P* = 0.03) between the STW Aware and Unaware groups. The percentage of participants with the age range from 20 to 40 years old was 78.9% (*N* = 228). In terms of education level, 33.6% had undergraduate degree, 51.9% had junior college and 14.5% had at least less than secondary school. Forty-seven percent of the participants were physician, 47.1% were nurse and 5.9% were others, such as laboratory technologist and imaging technologist. Among the professional title of participants, 71.3% were senior, 20.4% were mid-level, and 8.3% were junior. However, the age, education level, current profession and professional title displayed no difference between the two groups (Table 1).

### The knowledge of risk factors, symptoms, and acute management of stroke

When comparing the proportion of PHPs who knew the risk factors of stroke well in both groups, it showed no significant differences. The proportion of accurate perceptions about the target of < 140/90 mmHg for secondary prevention of stroke was significantly higher in the STW Aware group than that in the Unaware group (40 vs. 27.0%, *P* < 0.05). While the proportion of accurate perceptions about the target of < 7.0 mmol/L for fasting blood glucose control showed no significantly difference between two groups (Table 2). The proportion of PHPs who were able to recognize seven symptoms of stroke shown no difference between the STW Aware group and Unaware group, expect the symptom of loss of consciousness (75.7 vs. 62.6%, *P* < 0.05). The proportion of accurate perceptions about the F.A.S.T. Stroke Assessment also shown no difference between two groups (Table 3). A higher proportion of PHPs in the STW Aware group believed that intravenous thrombolysis was an effective treatment for acute ischemic stroke (96.5 vs. 79.9%, *P* < 0.01). The recommendations that endovascular therapy is indicated for acute ischemic stroke was perceived by a higher proportion of PHPs in the STW Aware group than that in the Unaware group (62.6 vs. 6.9%, *P* < 0.01). However, the knowledge of pre-hospital management showed no significant difference between the STW Aware group and the Unaware group (Table 4).

**Table 2:** The knowledge and awareness among primary healthcare providers regarding risk factors of stroke.

**Items**	**All (*n* = 289)**	**STW Aware group (*n* = 115)**	**STW Unaware group (*n* = 174)**	**χ^2^**	***P*** **-value**
**Risk factor for stroke**, ***n*** **(%)**					
Hypertension	259 (89.6)	102 (88.7)	157 (90.2)	0.2	0.4
High cholesterol levels	219 (75.8)	85 (73.9)	134 (77.0)	0.5	0.3
Diabetes mellitus	212 (73.4)	81 (70.4)	131 (75.3)	0.8	0.2
Obesity	211 (73.0)	80 (69.6)	131 (75.3)	0.9	0.2
Smoking	272 (94.1)	110 (95.7)	162 (93.1)	0.7	0.3
Alcohol consumption	216 (74.7)	92 (80.0)	124 (71.3)	1.5	0.1
**Target of**<**140/90 mmHg for secondary prevention of stroke, n (%)**				4.8	0.03
Yes	93 (32.2)	46 (40.0)	47 (27.0)		
No	196 (67.8)	69 (60.0)	127 (73.0)		
**Target of**<**7.0 mmol/L for fasting blood glucose control**, ***n*** **(%)**				3.3	0.1
Yes	137 (47.4)	47 (40.9)	90 (51.7)		
No	152 (52.6)	68 (59.1)	84 (48.3)		

STW, stroke treatment window.

**Table 3:** The knowledge and awareness among primary healthcare providers regarding symptoms of stroke.

**Items**	**All (*n* = 289)**	**STW Aware group (*n* = 115)**	**STW Unaware group (*n* = 174)**	**χ^2^**	***P*** **-value**
**Stroke symptoms**, ***n*** **(%)**					
Weakness on one body side	231 (79.9)	94 (81.7)	137 (78.7)	0.5	0.3
Face weakness	236 (81.7)	97 (84.4)	139 (79.9)	0.8	0.2
Speech impairment	233 (80.6)	95 (82.6)	138 (79.3)	0.5	0.3
Impairment of balance	217 (75.1)	88 (76.5)	129 (74.1)	0.3	0.4
Headache	198 (68.5)	83 (72.2)	115 (66.1)	1.0	0.2
Visual impairment	216 (74.7)	86 (74.8)	130 (74.7)	−0.1	0.5
Loss of consciousness	196 (67.8)	87 (75.7)	109 (62.6)	2.2	0.01
**F.A.S.T. stroke assessment**, ***n*** **(%)**					
Yes	236 (81.7)	97 (84.4)	139 (79.9)	0.9	0.3
No	53 (18.3)	18 (15.7)	35 (20.1)		

STW, stroke treatment window.

**Table 4:** The knowledge and awareness among primary healthcare providers regarding stroke treatment and the response when stroke was suspected.

**Items**	**All (*n* = 289)**	**STW Aware group (*n* = 115)**	**STW Unaware group (*n* = 174)**	**χ^2^**	***P*** **-value**
**Best period for stroke treatment is**<**4.5 h**, ***n*** **(%)**				284.8	<0.01
Yes	115 (39.8)	115 (100)	0 (0)		
No	174 (60.2)	0 (0)	174 (100)		
**Thrombolytic therapy is indicated for acute ischemic stroke**, ***n*** **(%)**				15.0	<0.01
Yes	250 (86.5)	111 (96.5)	139 (79.9)		
No	39 (13.5)	4 (3.5)	35 (20.1)		
**Endovascular therapy is indicated for acute ischemic stroke**, ***n*** **(%)**				101.6	<0.01
Yes	84 (29.1)	72 (62.6)	12 (6.9)		
No	305 (70.9)	43 (37.4)	162 (93.1)		
**Pre-hospital management for patients with acute stroke**, ***n*** **(%)**					
Observation of circulatory/respiratory problems	273 (94.5)	110 (95.6)	163 (93.7)	0.5	0.5
Observation of the heart	241 (83.4)	99 (86.1)	142 (81.6)	1.0	0.3
Securing the airways	266 (92.0)	110 (95.7)	156 (89.7)	3.4	0.1
Establishing intravenous access	264 (91.3)	106 (92.2)	158 (90.8)	0.2	0.7
Oxygen inhalation	256 (88.6)	105 (91.3)	151 (86.8)	1.4	0.2
Assess for hypoglycemia	214 (74.1)	92 (80.0)	122 (70.1)	3.5	0.1
Transferring the patient to a nearby stroke center	244 (84.4)	101 (87.8)	143 (82.2)	1.7	0.2

STW, stroke treatment window.

### Training on stroke management

Among all subjects, 80% of PHPs in the STW Aware group reported attending training on stroke management compared with 58.1% in the Unaware group (*P* < 0.01). In both groups, most participants received 1–2 trainings per year at their local hospital, whereas in the STW Aware group, more PHPs received more than two trainings per year (*P* < 0.01). There were no significant differences in educational strategies for stroke management between the two groups (Table 5).

**Table 5:** The knowledge and awareness among primary healthcare providers regarding training of stroke management.

**Items**	**All (*n* = 289)**	**STW Aware group (*n* = 115)**	**STW Unaware group (*n* = 174)**	**χ^2^**	***P*** **-value**
**Received training on stroke management**, ***n*** **(%)**				14.1	<0.01
Yes	193 (67.8)	92 (80.0)	101 (58.1)		
No	96 (33.2)	23 (20.0)	73 (42.0)		
**Times of training on stroke management per year in local hospital**, ***n*** **(%)**				28.5	<0.01
1–2	188 (65.1)	61 (53.0)	127 (73.0)		
3–4	48 (16.6)	17 (14.8)	31 (17.8)		
5–6	32 (11.1)	25 (21.7)	7 (4.0)		
7–8	9 (3.1)	5 (4.4)	4 (2.3)		
9–10	1 (0.4)	0 (0)	1 (0.6)		
>10	11 (3.8)	7 (6.1)	4 (2.3)		
**Education strategies for stroke management**, ***n*** **(%)**				3.7	0.3
Lectures or courses	149 (51.6)	64 (55.7)	85 (48.9)		
Video-based education	83 (28.7)	34 (29.6)	49 (28.2)		
Stroke resources shared on the web or social media	35 (12.1)	9 (7.8)	26 (14.9)		
Stroke simulation training	22 (7.6)	8 (7.0)	14 (8.0)		

STW, stroke treatment window.

### Predictors of stroke knowledge and awareness among primary healthcare providers

Multiple logistic regression was performed to examine the predictors of stroke knowledge and awareness among PHPs (Table 6). The results showed that the predictors included sex (OR: 2.3, 95% CI, 1.2–4.6), received training on stroke management (OR: 2.9, 95% CI, 1.6–5.1), and times of training on stroke management per year in local hospital (OR: 0.7, 95% CI, 0.6–0.9).

**Table 6:** Predictors of stroke knowledge and awareness among primary healthcare providers.

**Predictors**	***P*** **-value**	**Odds ratio**	**95% confidence interval**
Sex	0.02	2.3	1.2–4.6
Received training on stroke management	<0.01	2.9	1.6–5.1
Times of training on stroke management per year in local hospital	<0.01	0.7	0.6–0.9

## Discussion

Acute stroke care is an emergency that is conceptualized as a stroke survival chain occurring between stroke onset and appropriate disposition of the acute stroke patient receiving reperfusion therapy, and includes the eight steps of acute stroke recognition, diagnosis, and treatment (6, 16). Delays in any step can lead to delays in overall treatment and may even make the patient ineligible for certain reperfusion therapies (16). Especially in geographic areas with limited stroke centers, PHPs can be reconceptualized as the backbone of the stroke survival chain in some low- and middle-income countries or regions (16, 17). In the present cross-sectional survey about assessment of stroke knowledge and awareness among PHPs from the KQI-ASC Project, we found that only 115 (39.7%) participants aware that the optimal time window for IVT in acute stroke is less than 4.5 h. Gender of PHPs, stroke management training received, and number of stroke management presentations given at local hospitals per year were the predictors of stroke knowledge and awareness among PHPs.

Geographic level, hospital level, and healthcare provider level play a role in timely access to advanced healthcare services, including emergency stroke care services, to prevent unnecessary mortality and morbidity (17–20). In a report by Yang et al., only 49.1% of 997 community healthcare practitioners were aware of the time window for stroke management in 9 community health centers in Guangdong province, southern China, which was higher than what we found in Kezhou, northwest China, probably because Guangdong was a developed coastal region of China with a higher level of stroke care among PHPs than other areas in China (11). In the United States, there are also regional and hospital-level variabilities in stroke care. Zachrison et al. described the ability of community emergency departments (EDs) to diagnose and treat acute stroke and found that of 154 participating EDs in 30 states, 65 reported having written protocols for managing acute stroke (17). Khettar et al. conducted a cross-sectional study by administering an electronic standardized self-assessment questionnaire to community pharmacists in the Rhône-Alpes region of France and showed that 104 participants presented moderate levels of stroke management knowledge (20). Thus, even in a high-functioning pre-hospital stroke care system, optimizing the assessment and treatment of stroke patients in primary healthcare practitioners is necessary to strengthen the entire stroke survivorship chain (16).

Lack of awareness of stroke risk factors, symptoms, and insufficient attention to thrombolytic therapy in PHPs may lead to pre-hospital delays. We revealed that 40% of PHPs in the STW Aware group were familiar with the secondary stroke prevention goal of < 140/90 mmHg, compared with 27% in the Unaware group. In addition, our findings showed that PHPs were not sufficiently aware of stroke symptoms, with only 62.6% of PHPs knowing that loss of consciousness is also a symptom of stroke, which is consistent with previous findings (11). We also indicated that 79.9% of PHPs had the knowledge that AIS was amenable to intravenous thrombolysis within 4.5 h, but only 6.9% of PHPs knew that AIS was also amenable to endovascular treatment in the STW Unaware group, and these proportions were lower than in the STW Aware group. Therefore, training of PHPs in acute stroke management becomes urgent. In our study, only 58.1% of PHPs reported attending training on stroke management in the STW Unaware group and most of them received less than two trainings per year. This was similar to what has been found by Yang et al., where only 23.5% of community healthcare providers in the unawareness group reported participation in training on cerebrovascular disease management (11). Multiple logistic regression analysis also revealed an association between female gender, received training, times of training and the knowledge and awareness on stroke management.

One of the most important goals in improving the quality of treatment for AIS is to reduce door-to-needle time (DTN) and door-to-penetration (DTP) time. The AHA recommends a DTN time of less than 60 min and a DTP time of less than 90 min (19). Various training programs have been designed to improve the knowledge of first responders in order to improve the quality of emergency care for stroke patients in China and other countries and to promote the further development of the emergency training system (21–24). A pre-hospital EMSs training program consisting of three courses combining video learning, group discussions and quizzes was implemented in Shanghai, China. The results showed that the average DNT after the training was 53 min compared to 84.0 min before the training, demonstrating a significant reduction (21). Rababah et al. designed a randomized post-test study that consisted of a one-session stroke education program offered to 189 healthcare providers (physicians, registered nurses, and paramedics) in acute care settings, and the educational program implemented had a positive effect on improving healthcare providers' knowledge of stroke (22). However, some studies suggest that future assessments need to consider more sustained and multisite efforts supported by implementation science methods, rather than just DTN or DTP time, such as the patient's outcomes (23). Therefore, we developed a simulation-based education curriculum to train PHPs in stroke management according to Kern's six-step approach (25), and the Kirkpatrick model was used to guide the evaluation of intervention effects (26). Because only 33.6% of PHPs in our sample had undergraduate degree, a curriculum on pre-hospital and in-hospital management of stroke was designed to accommodate the needs of primary care providers, following the Chinese guideline (15).

The present study has several limitations. First, this is a cross-sectional survey and there is selection bias in our sample of PHPs who participated in the KQI-ASC Project in northwest China. These PHPs have high participation and are likely to be high performers and may not be representative of all PHPs. Consequently, the results may not be generalizable to other settings. Second, our data are self-reported and therefore depend on the knowledge of the respondents and may be influenced by social desirability bias (17). Finally, essential conditions for an effective stroke survival chain include a low threshold for detection, rapid onsite assessment of cardinal symptoms, and immediate transport by ambulance of patients considered for thrombolysis (27). Only a fraction of stroke patients is willing to seek PHPs rather than EMS (8). Therefore, further investigation of stroke knowledge and awareness among other providers of stroke EMS is warranted (28).

In conclusion, PHPs present with a mild to moderate level of stroke management knowledge in northwest China. PHPs in Kezhou need to improve their ability to deal with acute stroke onset in a timely and effective manner and provide effective treatment within a favorable “stroke treatment window”. To help improve the stroke related health services system, continuing education strategies that contribute to increased stroke knowledge and awareness among PHPs should be considered.

## Data availability statement

The original contributions presented in the study are included in the article/supplementary material, further inquiries can be directed to the corresponding author.

## Ethics statement

The studies involving human participants were reviewed and approved by Institutional Review Board of the Affiliated Kezhou People's Hospital of Nanjing Medical University. The patients/participants provided their written informed consent to participate in this study.

## Author contributions

G-BD and Y-FW drafted the manuscript. QS, H-JH, and X-MW added the survey data. QS and Y-FW revised the manuscript. All authors contributed to the article and approved the submitted version.

## Footnotes

^1^*Kizilsu Kirgiz Autonomous Prefecture People's Government*. Available online at: https://www.xjkz.gov.cn/xjkz/c101655/202108/14c0526af0454fb0a46f625868d0e452.shtml (accessed January 30, 2023).

^2^*Kizilsu Kirgiz Autonomous Prefecture People's Government*. Available online at: https://www.xjkz.gov.cn/xjkz/c101718/202207/22fba6258e93455a823d893cabf9840d.shtml (accessed January 30, 2023).
